# Clinicopathological and genomic analysis of SWI/SNF chromatin remodeling abnormalities with a focus on SMARCA4 in cancer of unknown primary

**DOI:** 10.1007/s00432-025-06293-9

**Published:** 2025-08-28

**Authors:** Yasutaka Tono, Koshi Sukeno, Akira Tsunoda, Mariko Okayama, Hiroki Oka, Hiroyasu Oda, Kanako Saito, Yoshiki Yamashita, Masayasu Taniguchi, Makoto Ikejiri, Satoshi Tamaru, Masaki Tanabe, Hiroshi Imai, Masatoshi Watanabe, Toshiro Mizuno

**Affiliations:** 1 Department of Medical Oncology, Mie University Hospital, 2-174, Tsu, 514- 8507 Mie Japan; 2 Department of Medical Oncology, Ise Red Cross Hospital, Mie 516-8512 Ise, Japan; 3 Department of Clinical Laboratory, Mie University Hospital, Tsu, 514-8507 Japan; 4 Clinical Research Support Center, Mie University Hospital, Tsu, 514-8507 Japan; 5 Pathology Division, Mie University Hospital, Tsu, 514-8507 Mie Japan; 6 Department of Oncologic Pathology, Mie University Graduate School of Medicine, Tsu, 514-8507 Mie Japan

**Keywords:** Cancer of unknown primary, SMARCA4, SWI/SNF, Chromatin remodeling

## Abstract

**Purpose:**

The estimation of the primary site is crucial when considering chemotherapy regimens in cancer of unknown primary (CUP). The task is particularly challenging for poorly differentiated or undifferentiated carcinoma, or unknown histological tumors with unknown primary (U-CUP). Instead of site-specific chemotherapy, a biomarker-guided therapy using genomic testing is required to predict the efficacy of molecular-targeted agents and immune checkpoint inhibitors (ICI). We focused on inactivating the SWI/SNF complex, a chromatin regulatory complex. We investigated the clinical features of CUP with SWI/SNF chromatin remodeling abnormalities and examined whether SWI/SNF chromatin remodeling abnormalities are a predictive marker of ICI efficacy.

**Methods:**

A multi-institutional observational study was conducted between January 2009 and March 2022. Immunostaining for SMARCA2, SMARCA4, and SMARCB1 was performed on 80 patients with CUP. Nextgeneration sequencing analysis was conducted on *SMARCA4*,* SMARCA2*,* SMARCB1*,* ARID1A*,* PBRM1*,* ARID2*, and *ARID1B*, which are frequent SWI/SNF-associated genes, in 32 patients with CUP.

**Results:**

Immunohistochemistry revealed that the loss of SMARCA4 protein was most frequent, occurring in 14 patients (17.5%). Among the 32 patients with CUP, SMARCA4 mutations were detected in 50% (*n* = 16) of patients. In 6 cases with truncating mutations, immunostaining revealed protein loss. U-CUP cases were associated with loss of SMARCA4 protein. In SMARCA4-deficient patients, overall survival was prolonged in the ICI-containing regimen group (*p* = 0.033).

**Conclusion:**

This study demonstrated SWI/SNF chromatin remodeling abnormalities in CUP and the association between SMARCA4 deficiency and U-CUP. It suggests a potential strategy for selecting an ICI regimen for CUP, particularly U-CUP, with SMARCA4 deficiency.

**Supplementary Information:**

The online version contains supplementary material available at 10.1007/s00432-025-06293-9.

## Introduction

Cancer of unknown primary (CUP) is a malignancy characterized by metastatic disease in which the primary site remains unidentified despite thorough investigation (Pavlidis et al. 2003). Approximately 20% of CUP cases are classified as favorable subsets, where treatment is guided by clinical characteristics, disease distribution, and histology. For example, elevated serum CA 125 levels, peritoneal metastasis, and serous adenocarcinoma are used to guide treatment strategies similar to those for stage III ovarian cancer. The remaining 80% of CUP cases are considered unfavorable subsets with a poor prognosis, typically treated with an empirical platinum-based regimen or site-specific therapy, presumed based on clinical findings (Pavlidis and Pentheroudakis 2012; Hasegawa et al. 2018).

Recent studies suggest that molecular profiling, including multi-gene expression assays, may improve outcomes in unfavorable subsets of CUP by guiding site-specific, targeted, or immune-based therapies. These assays help identify putative primary sites and actionable alterations (Wasan et al. 2023; Liu et al. 2024). Van Mourik et al. similarly reported improved survival with genomically informed treatment strategies (van Mourik et al. 2023). However, presuming the primary site followed by site-specific therapy has some problems. It is particularly challenging in cases of poorly differentiated or undifferentiated carcinoma, or tumors of unknown histological origin, as the organ of origin is often not identifiable histologically, and organ-specific immunostaining may be negative. We categorized tumors of unknown primary that are undifferentiated and cannot be classified into specific histologic subtypes as U-CUP. The contribution of genomic findings for the assessment of origin in U-CUP is limited. Therefore, other clinical approaches are necessary to determine optimal therapeutic strategies.

Undifferentiated tumors are often switch/sucrose non-fermenting (SWI/SNF)-deficient, characterized by aggressive malignancies with undifferentiated or rhabdoid cells that are resistant to chemotherapy, progress rapidly, and have a poor prognosis (Sauter et al. 2017; Yoshida et al. 2017). SWI/SNF is the most frequently mutated chromatin regulatory complex in human cancers and exhibits a mutation pattern similar to that of *TP53* (Kadoch et al. 2013). These tumors have loss-of-function mutations in genes encoding SWI/SNF complex proteins, which are involved in chromatin remodeling (Oike et al. 2013). Key subunit component genes include *SMARCA4 (BRG1), SMARCA2 (BRM),* and *SMARCB1 (INI1 (BAF47)*. Tumors caused by mutations in these genes include SMARCA4-deficient thoracic sarcoma, small cell carcinoma of the ovarian hypercalcemic type, and *SMARCB1*-deficient malignant rhabdoid tumors. SWI/SNF gene inactivation has been reported in various cancers, including lung, gastric, and pancreatic cancers (Oike et al. 2013).

Clinical features of SWI/SNF-deficient tumors, including SMARCA4-deficient tumors, have been observed in U-CUP. Therefore, therapeutic agents for SWI/SNF-deficient tumors may be effective for treating U-CUP. SWI/SNF gene alterations are associated with more favorable clinical outcomes in patients treated with immune checkpoint inhibitors (ICI), and these alterations may serve as predictive markers for ICI therapy in multiple cancers (Wang et al. 2023). In particular, recent studies have reported that SMARCA4-deficient non-small cell lung cancers respond favorably to ICIs. Given the morphological and molecular similarities between SMARCA4-deficient thoracic tumors and U-CUP, we hypothesized that SMARCA4 deficiency may similarly predict ICI efficacy in CUP. The CUPISCO trial, which compared comprehensive genomic profiling (CGP)-guided targeted therapy or immunotherapy with platinum-based chemotherapy for CUP, showed that molecular-guided therapy resulted in better progression-free survival (PFS) than chemotherapy (Westphalen et al. 2023; Kramer et al. 2024). However, molecular targeted agents have not been recommended for chromatin remodeling-related genes in CGP, such as *SMARCA4.* Further, to the best of our knowledge, no studies have examined whether SWI/SNF chromatin remodeling abnormalities in CUP predict ICI efficacy.

In this study, we investigated the pathological and genetic SWI/SNF chromatin remodeling abnormalities associated with SWI/SNF in CUP, particularly U-CUP, and explored its clinical characteristics. We aimed to determine whether SWI/SNF chromatin remodeling abnormalities can predict the efficacy of ICI in patients with CUP.

## Methods

### Patients

A multi-institutional observational study was conducted to investigate SWI/SNF chromatin-remodeling abnormalities in patients with CUP. This study enrolled 80 patients treated at Mie University Hospital and Ise Red Cross Hospital in Japan between January 2009 and March 2022. Clinical information was obtained from patient records. Patients with characteristics such as female sex, adenocarcinoma, and axillary lymph node metastasis only received surgery-based treatment and were managed as breast cancer, as other primary tumors were considered less likely to be clinically significant. They were typically included in the good prognosis group in CUP. Due to a previous report highlighting the rarity of abnormal chromatin remodeling in this subset (Schwartz et al. 2021), they were excluded from the study. Among favorable CUP subsets, cases fulfilling the criteria for single metastatic deposit or oligometastatic disease amenable to local ablative treatment, head and neck-like CUP (squamous cell carcinoma involving non-supraclavicular cervical lymph nodes), and colon-like CUP (adenocarcinoma with CK7-negative, CK20- and CDX2-positive immunophenotype) were included in this study. Clinical data such as age, sex, presumed primary site, metastatic location, pathology, and laboratory findings were collected, along with information on the chemotherapy regimen, PFS, and overall survival (OS). Data were obtained from the records of Mie University Hospital and Ise Red Cross Hospital in accordance with the principles outlined in the Declaration of Helsinki. This study was approved by the Institutional Review Board. An opt-out approach was employed on the official website of each institute to allow patients to decline participation by requesting the provided contact information.

### Patient selection and tissue availability

In this study, all included patients underwent immunohistochemistry (IHC) analysis. As IHC requires a minimal amount of tissue, cases were first screened by a pathologist to confirm sufficient sample availability for IHC. Patients with inadequate tissue for IHC were excluded from the study. In cases where sufficient residual formalin-fixed paraffin-embedded (FFPE) tissue remained after IHC analysis, next-generation sequencing (NGS) was also performed. Due to limited specimen quantity, NGS could not be performed in all cases. No patient underwent NGS without prior IHC analysis.

### Immunohistochemical analysis

Tumors that are deficient in SMARCA4, SMARCA2, or SMARCB1 are characteristic of those lacking functional SWI/SNF chromatin remodeling complexes; thus, immunohistochemistry for these proteins was performed. In this study, SMARCA2, SMARCA4, and SMARCB1 positivity were evaluated using a cutoff of 10% by visual assessment. Formalin-fixed paraffin-embedded blocks were sectioned at 4-µm thickness and subjected to immunostaining. The following primary antibodies were used: rabbit anti-human SMARCA2 antibody (Sigma Aldrich, St. Louis, MO, USA), rabbit anti-mouse BAF47 antibody (Becton Dickinson, Franklin Lakes, NJ, USA), and rabbit anti-mouse BRG1 antibody (Abcam, Cambridge, UK).

As sarcomas require specific chemotherapy, they should be excluded from CUP. However, tumors with epithelioid morphology that may possibly represent sarcoma could have been diagnosed as U-CUP due to non-specific immunoprofiles and uncertain histogenesis.

Claudin-4 is expressed in most epithelial cells and carcinomas. Previously, Claudin-4 expression has been reported in SWI/SNF complex-deficient undifferentiated carcinomas showing loss of SMARCB1, SMARCA4, or ARID1A; immunostaining for Claudin-4 can differentiate SWI/SNF complex-deficient undifferentiated carcinomas from sarcomas with epithelioid morphology (Schaefer et al. 2017). The assay was performed using mouse anti-human Claudin-4 antibody (Invitrogen, Carlsbad, CA, USA). Claudin-4 immunohistochemistry was performed in SMARCA4-deficient U-CUP cases to confirm epithelial differentiation and exclude the possibility of sarcoma with epithelioid morphology. The Claudin-4 staining results were not used to reclassify cases or incorporated into the primary outcome analyses but served as a supplementary pathological assessment.

### DNA extraction

Genomic DNA was extracted from formalin-fixed paraffin-embedded (FFPE) tissue samples. Fifteen sections of 10 µm thickness were prepared using the FFPE tissue block. A pathologist manually confirmed that the proportion of tumor cells was greater than 20%. Genomic DNA was extracted using the QIAmp DNA FFPE Tissue Kit (Qiagen, Inc.), following the manufacturer's instructions. The quality and quantity of DNA samples were assessed using Qubit® dsDNA (Thermo Fisher Scientific, Inc.), according to the manufacturer's protocol.

### Next-generation sequencing (NGS)

NGS was performed in cases where sufficient tumor tissue was available for genomic testing. Importantly, no preselection based on immunohistochemistry or clinical suspicion of SWI/SNF abnormalities was performed before NGS testing. The selection was based solely on specimen availability, independent of clinical features or pathological findings suggestive of SWI/SNF alterations.

NGS of genomic DNA was performed using the Ion Ampliseq™ Oncomine Tumor Specific Panel (cat. no. A45045; Thermo Fisher Scientific), which examines up to 10 cancer-associated genes. Among approximately 30 SWI/SNF-related genes, we selected seven genes—*SMARCA4*, *SMARCA2*, *SMARCB1, ARID1A, PBRM1, ARID2,* and *ARID1B—*with particularly high frequencies in The Cancer Genome Atlas (TCGA) Pan-Cancer dataset (N = 10,967) and MSK-IMPACT (N = 10,945) (Centore et al. 2020).

Briefly, 10 ng of genomic DNA was used to construct barcoded DNA libraries using an Ion AmpliSeq Kit for Chef DL8 (cat. no. A29024; Thermo Fisher Scientific, Inc.). The libraries were sequenced using an Ion GeneStudio™ S5 platform (Thermo Fisher Scientific, Inc.). The sequencing was performed on a single end using the Ion 510™, Ion 520™, and the Ion 530™ Kit-Chef (A34461). The library loading concentration was 25 pM. Sequencing reads were aligned to the reference genome GRCh38 and converted into binary alignment and map files using Torrent Suite 5.12.3 software (Thermo Fisher Scientific, Inc.). Sequence variants were called using Ion Reporter™ 5.20 (Thermo Fisher Scientific, Inc.), according to the manufacturer's protocols. The mean read depth of coverage for DNA sequencing was > 1500-fold.

In this study, the term “SWI/SNF chromatin remodeling abnormalities” was used consistently to refer to abnormalities involving genes associated with the SWI/SNF chromatin remodeling complex.

“SMARCA4 deficiency” was defined as the loss of SMARCA4 protein expression detected by immunohistochemistry. We classified SMARCA4 genomic alterations into two categories: class 1 (truncating mutations), which were associated with loss of protein expression, and class 2 (missense mutations), in which protein expression was retained. Although immunohistochemistry does not always correlate perfectly with genomic alteration, we prioritized genomic sequencing results over immunohistochemical findings when assigning mutation classes. In cases where genomic alterations were unknown, classification was based on immunohistochemical results.

### Classification of oncogenic mutations and bioinformatics analysis

The impact of point mutations on protein function was assessed using PolyPhen2 (genetics.bwh.harvard.edu/pph2/index.shtml) (Adzhubei et al. 2010). Pathogenic mutations included point mutations leading to premature stop codons, small insertions or deletions causing frameshifts or premature stop codons, large rearrangements, and mutations affecting intron donor or acceptor splice sites, based on ACMG laboratory guidelines (Richards et al. 2015). Remaining variants were considered pathogenic based on functional analyses reported in the literature, identification as pathogenic or likely pathogenic in ClinVar (https://www.ncbi.nlm.nih.gov/clinvar/).

### Statistical analysis

The sample size for each statistical analysis was n = 80. Non-continuous data were compared using Fisher's exact test (two-sided), with *P* < 0.05 indicating statistical significance. The assumptions for Fisher's exact test were verified, including the independence of observations and the adequacy of the sample size. The alpha level for all tests was set at 0.05. Descriptive statistics were reported for all variables. Data from patients who were alive or lost to follow-up were censored on the last date of contact. The Kaplan–Meier method was used to estimate OS. The assumptions of the Kaplan–Meier method, including non-informative censoring and constant survival probabilities, were verified. All analyses were performed using IBM SPSS Statistics version 29 (IBM Japan, Ltd.). The actual P-value was reported for each test to provide precise information about the statistical significance of the results.

## Results

### Patient characteristics

The clinical characteristics of 80 patients with CUP are shown in Table 1. The median age was 67 years (range, 31–85 years). Thirty-one of the 80 patients (38.8%) were women. Histological types included adenocarcinoma (32 patients, 40%), squamous cell carcinoma (18 patients, 22.5%), poorly differentiated or undifferentiated carcinomas and malignant tumors (24 patients, 30%), neuroendocrine carcinoma (4 patients, 5%), and carcinosarcoma (2 patients, 2.5%). The most common metastatic sites were the lymph nodes (59 cases, 73.7%), bone (22 cases, 27.5%), lungs (13 cases, 16.2%), and liver (12 cases, 15.0%). Among patients classified as having metastases in only one organ (n = 34), 22 had lymph node-only metastases, two had bone-only metastases, three had peritoneal dissemination, six had soft tissue involvement, and one had pericardial metastasis. In terms of favorable CUP subsets, our cohort included seven cases of single-site/oligometastatic CUP, two head and neck-like CUP, and one colon-like CUP. Immunostaining revealed the loss of SMARCA2, SMARCA4, and SMARCB1 in five (6.2%), 14 (17.5%), and one (1.2%) of the 80 patients, respectively.

**Table 1:** Patient characteristics (*n* = 80)

	Patients (%) (*n* = 80)
Age, median (range)	67 (31–85)
*Gender*	
Male	49 (61.2)
Female	31 (38.8)
ECOG PS	
0–1	49 (61.2)
2	19 (23.8)
3–4	12 (15.0)
*Histology*	
Adenocarcinoma	32 (40.0)
Squamous cell carcinoma	18 (22.5)
Poorly/undifferentiated carcinoma or malignant tumor	24 (30.0)
Neuroendocrine carcinoma	4 (5.0)
Carcinosarcoma	2 (2.5)
*Number of organs with metastasis*	
1	34 (42.5)
2	19 (23.8)
3≦	27 (33.8)
*Metastatic sites*	
Lymph node	59 (73.7)
Bone	22 (27.5)
Lung	13 (16.2)
Liver	12 (15.0)
Peritoneum	11 (13.7)
Soft tissue	10 (12.5)
Adrenal gland	7 (8.7)
Pleura	5 (6.2)
Retroperitoneum	5 (6.2)
Ovary	4 (5.0)
Brain	4 (5.0)
Pericardium	3 (3.7)
Skin	3 (3.7)
Bone mallow	2 (2.5)
Others (Bowel, Kidney, Uterus, Spleen, Breast, Meninges, Parathyroid)	7 (8.7)
*Immunohistochemistry*	
SMARCA4 lost	14 (17.5)
SMARCA2 lost	5 (6.2)
SMARCB1 lost	1 (1.2)
Duration of follow-up (days), median (range)	258 (4-4990)

### Clinicopathologic and genomic status

**Fig. 1:**
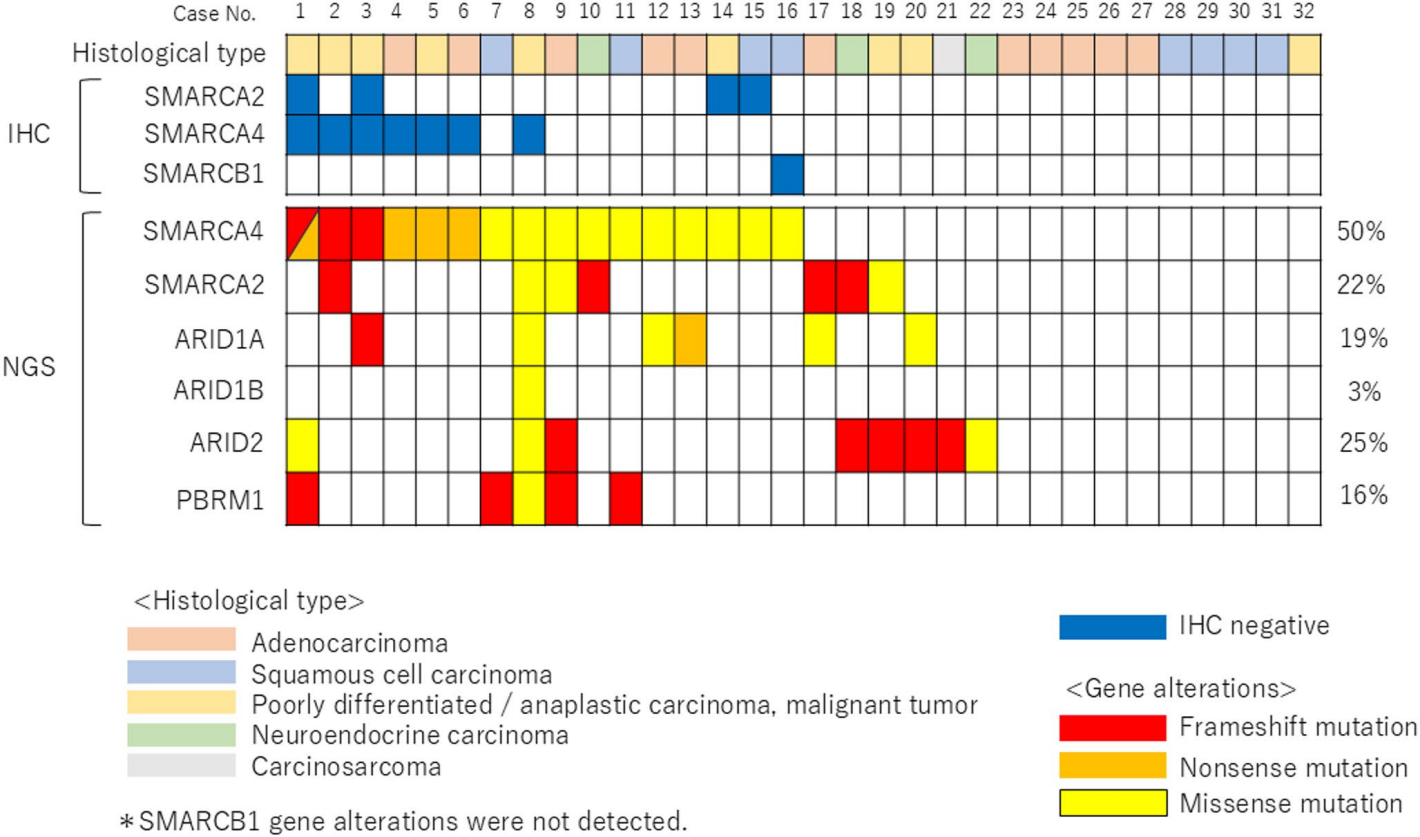
Histological type, immunohistochemistry, and chromatin remodeling-related genomic alterations in 32 patients with CUP with surplus specimens for NGS analysis. On the right, the frequency of copy number alterations is shown. Each column represents an individual case, aligned across panels to allow direct comparison of IHC and NGS findings

Histological type, immunohistochemical results, and genomic status are shown in Fig. 1. Among the 80 CUP cases, NGS could be performed in only 32 cases (40%) due to insufficient quantity or quality of tumor specimens. Protein loss of SMARCA2, SMARCA4, and SMARCB1 was observed in four (12.5%), seven (21.8%), and one (3.1%) cases, respectively (Fig. 1).

DNA analysis revealed SMARCA4 mutations in 16 cases (50%), ARID2 mutations in eight cases (25%), SMARCA2 mutations in seven cases (22%), ARID1A mutations in six cases (19%), PBRM1 mutations in five cases, and ARID1B mutations in one case (Fig. 1). No genomic alterations were observed in *SMARCB1*. As shown in Fig. 1, truncating mutations correlated with loss of SMARCA4 protein expression, while missense mutations were rarely associated with protein loss. Therefore, the IHC-only group may have been enriched with cases harboring non-truncating mutations not resulting in protein loss. Immunostaining loss of SMARCA2 and SMARCB1 did not correspond to gene abnormalities detected by NGS. SWI/SNF Chromatin remodeling abnormalities in CUP were more common in SMARCA4.

Two of the eight patients with U-CUP and SMARCA4 mutations (25%) tested negative for claudin-4. Both cases were positive for cytokeratin AE1/AE3. One case showed positivity for CK7 and CK20, while the other was negative for Desmin. These findings support the diagnosis of epithelial tumors rather than sarcomas.

### Association with clinical features and SWI/SNF chromatin remodeling abnormalities

We examined the clinical characteristics associated with SMARCA4 protein deficiency using immunohistochemical analysis (Table 2). No significant differences were observed in age, sex, PS, or the number of metastatic organs. SMARCA4 protein deficiency was more common in U-CUP (p = 0.072). Statistical analysis of clinical characteristics in patients with SMARCA4 gene abnormalities is shown in Table 3. Patients with SMARCA4 truncating mutations had undifferentiated histology compared to those with wild type (*p* = 0.073). There were no significant differences in age, sex, PS, or number of metastatic organs between the groups with or without SMARCA4 missense mutations.

**Table 2:** Clinical characteristics according to SMARCA4 protein loss in the immunohistochemical analysis

	All patients (n = 80) n (%)	Lost (n = 14) n (%)	SMARCA4 Retained (n = 66) n (%)	P value
Age, median (range)	67 (31–85)	67 (49–75)	67 (31–85)	
*Gender*				
Male	49 (61)	10 (71)	39 (59)	0.389
Female	31 (39)	4 (29)	27 (41)	
*ECOG PS*				
0–1	49 (59)	10 (71)	39 (59)	0.389
2–4	31 (41)	4 (29)	27 (41)	
*Poorly/Undifferentiated carcinoma or malignant tumor*				
Yes	24 (30)	7 (50)	17 (26)	0.072
No	56 (70)	7 (50)	49 (74)	
*Number of metastatic organ*				
1–2	53 (66)	9 (64)	44 (67)	0.864
3≦	27 (34)	5 (36)	22 (33)

**Table 3:** Clinical characteristics according to SMARCA4 gene mutation

	All patients (n = 32) n (%)	Truncating mutation (n = 6) n (%)	Missense mutation (n = 10) n (%)	Wild-type (n = 16) n (%)	P value
Age, median (range)	69 (48–82)	67 (49–71)	70 (49–80)	69 (50–82)	
*Gender*					
Male	20 (63)	4 (67)	6 (60)	10 (63)	1.000
Female	12 (37)	2 (33)	4 (40)	6 (37)	
*ECOG PS*					
0–1	19 (59)	3 (50)	7 (70)	9 (56)	0.712
2–4	13 (41)	3 (50)	3 (30)	7 (44)	
*Poorly/undifferentiated carcinoma or malignant tumor*					
Yes	9 (28)	4 (67)	2 (20)	3 (19)	0.073
No	23 (72)	2 (33)	8 (80)	13 (81)	
*Number of metastatic organs*					
1–2	20 (63)	3 (50)	7 (70)	10 (63)	0.797
3≦	12 (37)	3 (50)	3 (30)	6 (37)	

### Survival analysis

**Fig. 2:**
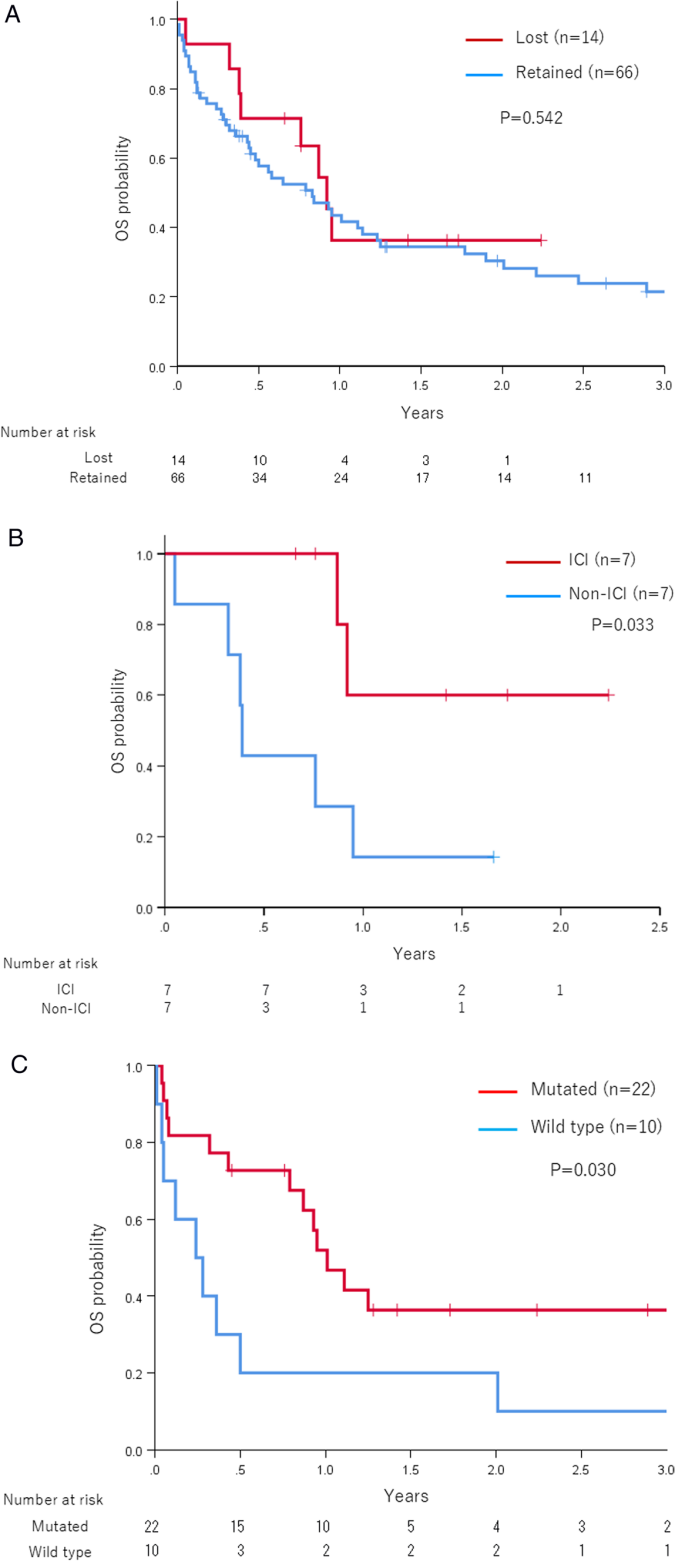
Overall survival of patients with CUP. **A** Stratified by immunohistochemical status of SMARCA4. **B** Stratified by ICI use among patients with SMARCA4 protein deficiency. **C** Stratified by genomic status of SWI/SNF chromatin remodeling abnormalities

We excluded SMARCA2 and SMARCB1 due to discordant immunohistochemical staining and genetic mutation results, focusing our survival analysis on the loss of SMARCA4 protein. There was no significant difference in OS between the SMARCA4-deficient and SMARCA4-retained groups (*p* = 0.542) (Fig. 2A). In patients with SMARCA4 protein deficiency, OS was significantly prolonged in the ICI-containing regimen group (*p* = 0.033) (Fig. 2B). In the SMARCA4-deficient group, six patients received ICI monotherapy, and one patient received an ICI combination regimen with cytotoxic chemotherapy. Among these seven patients, four received ICI as first-line treatment. Regarding biomarker status, PD-L1 expression was evaluated in two out of seven patients, and both showed PD-L1 positivity (≥ 1%). Tumor mutational burden (TMB) was assessed in five of seven patients, with two cases showing TMB ≥ 10 mutations per megabase. Microsatellite instability (MSI) status was assessed in five patients, and all were microsatellite stable (MSS) (Supplementary Table 1).

Subsequently, survival analysis was performed based on the DNA sequencing results of chromatin remodeling–related genes. Patients with CUP harboring alterations in chromatin remodeling genes had significantly longer OS than those without such alterations (*p* = 0.030) (Fig. 2C). However, log-rank tests for individual genes did not reveal any statistically significant differences.

Among the chromatin remodeling–related genes, *SMARCA4* was the most frequently altered. As protein expression can differ between truncating and missense mutations, subgroup analyses were conducted to assess whether the prognostic impact of ICI treatment varies according to the type of SMARCA4 mutation. Although no statistically significant differences in survival were observed between the two groups, patients with truncating mutations tended to exhibit improved survival compared to those with missense mutations following ICI administration. Supplementary Table 1 summarizes treatment response by mutation and ICI exposure; however, the analysis is limited by the small sample size. We additionally compared overall survival between patients with missense mutations according to the use of ICI-containing regimens. No significant difference in overall survival was observed between the ICI and non-ICI groups (*p* = 0.769; Supplementary Figure).

## Discussion

To our knowledge, this is the first report revealing the clinical features of CUP with SWI/SNF chromatin remodeling abnormalities using immunohistochemical and genomic analyses. Among the SWI/SNF complex-associated molecules identified, SMARCA4 protein deficiency and gene alterations were more common in the U-CUP group. Although SMARCA4 deficiency was not a prognostic factor, it may serve as a predictor of ICI efficacy. Therefore, the significance of this study lies in its suggestion that SMARCA4 truncating mutations, including in U-CUP cases—where treatment selection is particularly challenging—may serve as a potential biomarker for predicting response to ICI in CUP. Although nivolumab can be administered to patients with CUP regardless of PD-L1 expression in Japan, it is important to select patients with CUP based on such a biomarker.

Our study found that 17.5% of patients with CUP had SMARCA4 protein deficiency, and 50% had genetic abnormalities. In clinical findings, SMARCA4-deficient thoracic sarcoma or small-cell carcinoma of the ovarian hypercalcemic type were not observed in our cases; however, non-small cell lung cancer (NSCLC) was suspected in some cases. Notably, 80% of SMARCA4-deficient NSCLC are TTF-1 negative, occurring in approximately 5% of all NSCLC (Herpel et al. 2017). Research on SMARCA4 variants from targeted exome sequencing in various cancers found that missense mutations were most frequent overall, while truncating mutations were more common in NSCLC and CUP than in other tumors (Fernando et al. 2020). In NSCLC, truncating mutations are classified as class 1 and missense mutations as class 2, with loss of protein expression linked to class 1 mutations (Schoenfeld et al. 2020). Therefore, SMARCA4 protein expression is more likely to be defective in NSCLC and CUP cells compared to other cancers.

SMARCA4 abnormalities were more common in U-CUP than in other histological types. The association between SMARCA4 abnormalities and undifferentiated histology has been reported in other cancer types. Soft tissue neoplasms with SWI/SNF chromatin remodeling abnormalities often exhibit undifferentiated and rhabdoid cell morphology (Schaefer and Hornick 2021). Similar undifferentiated tumors with SMARCA4 deficiency have been reported in uterine endometrial carcinoma (Strehl et al. 2015). Undifferentiated pancreatic carcinomas show a higher frequency of abnormalities in SWI/SNF complex subunits, such as ARID1A and SMARCA4, and increased expression of EMT-related markers compared to ductal adenocarcinomas (Yamamoto et al. 2022). Although the association between SMARCA4 deficiency and tumor cell undifferentiation is not fully understood, our findings suggest that SMARCA4 deficiency may serve as a useful pathologic feature for identifying U-CUP.

Our exploratory analysis suggests that ICI may be effective for patients with SMARCA4 deficiency, specifically those with SMARCA4 truncating mutations. ICIs are expected to benefit SWI/SNF-related tumors (Naito et al. 2019). Significant correlations have been observed between alterations in the SWI/SNF signature and improved OS and PFS in ICI-treated patients across multiple cancers (Wang et al. 2023). Previous reports have demonstrated that alterations in SWI/SNF complex genes are closely associated with immunologically active tumor features, including high TMB, MSI-high status, and better response to ICIs in various cancers (Li et al. 2022). These findings suggest that SWI/SNF deficiency may enhance tumor immunogenicity and sensitize tumors to ICI therapy. Additionally, SMARCA4 abnormalities are linked to TMB-High and MSI-H (Peng et al. 2021). Treatment with ICIs has shown improved outcomes in SMARCA4-mutant NSCLC, with class 1 mutations responding best to ICIs (Schoenfeld et al. 2020). Our findings indicate that SMARCA4 truncating mutations may be predictive of ICI efficacy in CUP patients.

In our survival analysis, SMARCA4 gene abnormalities were not associated with prognosis. It has been reported many cancers with SWI/SNF chromatin remodeling abnormalities progress rapidly and have a poor prognosis (Sauter et al. 2017; Yoshida et al. 2017). Among tumors with SWI/SNF chromatin remodeling abnormalities, loss of SMARCA4 and SMARCA2 protein expression has been shown to be a poor prognostic factor, particularly in lung cancer (Reisman et al. 2003). The use of ICIs in our cases likely influenced the lack of difference in survival analysis.

This study had several limitations. As a small retrospective study, the results should be interpreted with caution. Limitations include the inclusion of neuroendocrine carcinoma, which is classified as CUP in Japanese guidelines (JSMO) but is excluded in ESMO guidelines. This may limit the comparability of our results with European CUP cohorts. Due to the small amount or poor quality of tissue samples, genomic analysis was feasible in less than half of the cases, limiting the robustness of genetic evaluation. We acknowledge that PD-L1 expression, TMB, and MSI status were not uniformly evaluated in all patients. Although PD-L1 positivity and high TMB were observed in a subset of cases, the limited and incomplete biomarker data preclude definitive conclusions. Therefore, we cannot rule out the possibility that these or other predictive biomarkers may have contributed to treatment efficacy, potentially confounding the observed association between SMARCA4 deficiency and ICI response.

Further research is needed to confirm our findings and to accumulate more cases of SWI/SNF-related genes.

## Conclusions

SWI/SNF chromatin remodeling abnormalities in CUP predominantly occur in SMARCA4. Class 1 mutations and SMARCA4 protein deficiency were more frequent in U-CUP. Given the variability in treatment lines, regimen types, and the small sample size, the efficacy of ICI in SMARCA4-deficient CUP remains a possibility rather than a definitive finding. Further accumulation of cases and more robust investigations are warranted to validate these observations. These findings underscore the need for further studies and analyses to validate the predictive value of SMARCA4 deficiency and explore additional SWI/SNF chromatin remodeling abnormalities. This study highlights the potential for personalized treatment strategies in CUP based on genomic and immunohistochemical profiling.

## Supplementary Information

Below is the link to the electronic supplementary material.

Supplementary Material 1

Supplementary Material 2

## Data Availability

The data produced and analyzed during the present study are available from the corresponding author on reasonable request.
