# Supplementary figures and images for: Clinicopathological and genomic analysis of SWI/SNF chromatin remodeling abnormalities with a focus on SMARCA4 in cancer of unknown primary
Source: J Cancer Res Clin Oncol. 2025 Aug 28;151(8):238. doi: 10.1007/s00432-025-06293-9 (PMC12390907; doi:10.1007/s00432-025-06293-9)

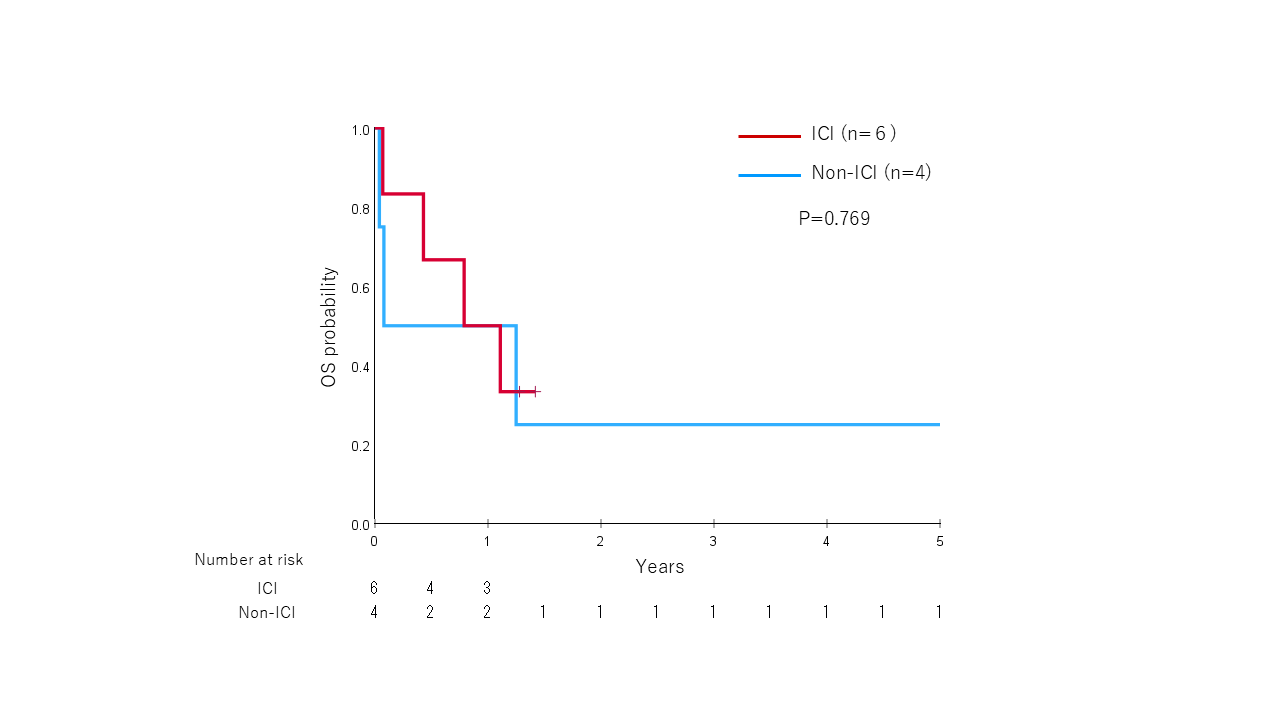

Supplement: Supplementary file 1 — Supplementary Material 1 [file 432_2025_6293_MOESM1_ESM.tif]
