# Supplementary material for: Clinicopathological and genomic analysis of SWI/SNF chromatin remodeling abnormalities with a focus on SMARCA4 in cancer of unknown primary
Source: J Cancer Res Clin Oncol. 2025 Aug 28;151(8):238. doi: 10.1007/s00432-025-06293-9 (PMC12390907; doi:10.1007/s00432-025-06293-9)
Supplement: Supplementary file 2 — Supplementary Material 2 [file 432_2025_6293_MOESM2_ESM.docx]

Supplementary Table 1.

1. SMARCA4 class1 mutation with ICI treatment

| **Age, Sex** | **SMARCA4**  **IHC** | **SMARCA4 alterations** | **ICI regimen** | **Combination partner** | **Line of ICI therapy** | **PD-L1**  **status** | **TMB** | **MSI** | **Response** | **PFS**  **(days)** |
| --- | --- | --- | --- | --- | --- | --- | --- | --- | --- | --- |
| 69F | lost | nonsense | Nivolumab | - | 1^st^-line | 1-5% | 7.6 | MSS | PD | 48 |
| 72M | lost | - | Atezolizumab | Carboplatin + Paclitaxel | 1^st^-line | - | - | - | PR | 581 |
| 50F | lost | - | Nivolumab | - | 1^st^-line | - | 23 | MSS | PR | 798 |
| 73M | lost | - | Nivolumab | - | 3^rd^-line | 1% | - | - | PD | 18 |
| 65M | lost | frameshift | Nivolumab | - | 2^nd^-line | - | 5 | MSS | PR | 1100 |
| 49M | lost | nonsense | Pembrolizumab | - | 1^st^-line | - | 10.9 | MSS | PR | 983 |
| 60M | lost | frameshift | Nivolumab | - | 2^nd^-line | - | 7.6 | MSS | PR | 112 |

1. SMARCA4 class2 mutation with ICI treatment

| **Age, Sex** | **SMARCA4**  **IHC** | **SMARCA4 alterations** | **ICI regimen** | **Combination partner** | **Line of ICI therapy** | **PD-L1 status** | **TMB** | **MSI** | **Response** | **PFS**  **(days)** |
| --- | --- | --- | --- | --- | --- | --- | --- | --- | --- | --- |
| 80F | retained | missense | Nivolumab | - | 2^nd^-line | - | - | - | SD | 88 |
| 72M | lost | missense | Atezolizumab | Carboplatin + Paclitaxel | 1^st^-line | - | - | - | PR | 574 |
| 75M | retained | missense | Nivolumab | Oral 5FU  + Oxaliplatin | 1^st^-line | 5%≦ | - | - | NA | 24 |
| 59F | retained | missense | Nivolumab | - | 2^nd^-line | - | - | - | PD | 23 |
| 48M | retained | missense | Nivolumab | - | 2^nd^-line | - | - | - | PD | 52 |
| 79F | retained | missense | Nivolumab | - | 2^nd^-line | - | - | - | CR | 793 |
